# Explanation of hand, foot, and mouth disease cases in Japan using Google Trends before and during the COVID-19: infodemiology study

**DOI:** 10.1186/s12879-022-07790-9

**Published:** 2022-10-29

**Authors:** Qian Niu, Junyu Liu, Zixi Zhao, Miyu Onishi, Asuka Kawaguchi, Anuradhi Bandara, Keiko Harada, Tomoki Aoyama, Momoko Nagai-Tanima

**Affiliations:** 1 Department of Human Health Sciences, Graduate School of Medicine, Kyoto University, 53, Kawahara-Cho, Shogoin, Sakyo-Ku, Kyoto, 606-8507 Japan; 2 Department of Intelligence Science and Technology, Graduate School of Informatics, Kyoto University, Kyoto, Japan

**Keywords:** HFMD, Infodemiology, Google Trends, Infection, Explanation

## Abstract

**Background:**

Coronavirus Disease 2019 (COVID-19) pandemic affects common diseases, but its impact on hand, foot, and mouth disease (HFMD) is unclear. Google Trends data is beneficial for approximate real-time statistics and because of ease in access, is expected to be used for infection explanation from an information-seeking behavior perspective. We aimed to explain HFMD cases before and during COVID-19 using Google Trends.

**Methods:**

HFMD cases were obtained from the National Institute of Infectious Diseases, and Google search data from 2009 to 2021 in Japan were downloaded from Google Trends. Pearson correlation coefficients were calculated between HFMD cases and the search topic “HFMD” from 2009 to 2021. Japanese tweets containing “HFMD” were retrieved to select search terms for further analysis. Search terms with counts larger than 1000 and belonging to ranges of infection sources, susceptible sites, susceptible populations, symptoms, treatment, preventive measures, and identified diseases were retained. Cross-correlation analyses were conducted to detect lag changes between HFMD cases and search terms before and during the COVID-19 pandemic. Multiple linear regressions with backward elimination processing were used to identify the most significant terms for HFMD explanation.

**Results:**

HFMD cases and Google search volume peaked around July in most years, excluding 2020 and 2021. The search topic “HFMD” presented strong correlations with HFMD cases, except in 2020 when the COVID-19 outbreak occurred. In addition, the differences in lags for 73 (72.3%) search terms were negative, which might indicate increasing public awareness of HFMD infections during the COVID-19 pandemic. The results of multiple linear regression demonstrated that significant search terms contained the same meanings but expanded informative search content during the COVID-19 pandemic.

**Conclusions:**

The significant terms for the explanation of HFMD cases before and during COVID-19 were different. Awareness of HFMD infections in Japan may have improved during the COVID-19 pandemic. Continuous monitoring is important to promote public health and prevent resurgence. The public interest reflected in information-seeking behavior can be helpful for public health surveillance.

**Supplementary Information:**

The online version contains supplementary material available at 10.1186/s12879-022-07790-9.

## Background

Hand, foot, and mouth disease (HFMD) is an infectious disease that causes blistering rashes on the mouth, hands, and feet. Most infected individuals recover from HFMD within a few days of infection. Various comorbidities, including myocarditis, neurogenic pulmonary edema, acute flaccid paralysis, and central nervous system complications such as meningitis, cerebellar ataxia, and encephalitis, can also occur [1, 2]. HFMD is distributed worldwide, and outbreaks often occur during the summer and early fall in the United States. Large outbreaks in Cambodia, China, Japan, Korea, Malaysia, Singapore, Thailand, and Vietnam have been reported over the past 2 decades [3, 4]. HFMD is seasonal in temperate Asia with a summer peak, and subtropical Asia with spring and fall peaks, but not in tropical Asia, indicating that temperate Japan has a climatic role [5]. During the summer of 2011, Japan had the largest HFMD epidemic on record, with 347,362 reported cases [6]. Coxsackievirus A6 (CV-A6) infection is responsible for most cases, with co-circulation of coxsackievirus A16 (CV-A16) and enterovirus A71 (EV-A71) [7]. EV-A71 was sporadically detected from October 2014 onward. It became the predominant serotype in 2018, with approximately 70,000 reported cases, following an increase in its spread from the end of 2017 [8]. Since June 2019, a severe outbreak of HFMD has occurred in multiple regions of Japan, attracting public attention [9]. As enteroviruses can spread rapidly by droplet and fomite transmission among children in daycare centers and kindergartens, understanding HFMD outbreaks is vital to public health, particularly during the Coronavirus Disease 2019 (COVID-19) pandemic [10].

Rapid recognition and reporting of HFMD infection are essential, and several studies have constructed models for explaining HFMD infection [11–15]. Rui et al. explored epidemiological characteristics and calculated the early warning signals of HFMD using a logistic differential equation (LDE) model in seven regions of China [11]. Yu et al. forecasted the number of HFMD cases with wavelet-based hybrid models in Zhengzhou, China [12]. Zhang et al. proposed a landscape dynamic network marker (L-DNM) to detect pre-outbreak signals of HFMD in Tokyo, Hokkaido, and Osaka, Japan [13]. Gao et al. used monthly HFMD infection cases and meteorological data to construct a weather-based early warning model with a generalized additive model across China [14]. Zhao et al. used a meta-learning framework and combines Baidu search queries for real-time estimation of HFMD cases [15]. The above studies used a range of data, including monthly or weekly HFMD infectious cases [11, 12, 14], dynamic information from city networks, horizontal high-dimensional data, records of clinic visits [13], meteorological data [14], and Baidu search queries data [15] which are relatively difficult to access or delayed updates in Japan. Traditional surveillance and reporting systems lag an outbreak by 1 to 2 weeks because of the reporting and verification process. In Japan, the National Institute of Infectious Disease (NIID) has monitored the outbreak of various infectious diseases and issued weekly reports since 1999, but delayed for several weeks [16]. In addition, no studies have focused on changes in HFMD affected by the COVID-19 pandemic by Internet searching data compared with previous studies. As of August 2022, Google search data was considered reliable because its market share in Japan has been over 70% since 2009 [17, 18]. Google Trends data offers real-time and labor-saving information search by the public, which may be valuable for infection surveillance.

The science of distribution and determinants of information in an electronic medium, specifically the Internet, or in a population, to inform public health and policy is defined as “infodemiology” [19]. Google Trends is frequently used in infodemiology research to gauge public interests [20]. Google Trends reflects public information-seeking behavior and allows users to analyze Google search data for specific search terms in any country or region over a selected period [21, 22]. Studies have shown that online query trends correlate with real-life epidemiologic phenomena such as flu [23], sinusitis [24], lifestyle-related disease [25], asthma [26], and pruritus [27]. Researchers have also investigated public interest and information-seeking behaviors in chronic obstructive pulmonary disease (COPD) [28], cancer [29, 30], bariatric surgery [31], kidney stone surgery [32], and suicide [33]. During the COVID-19 pandemic, similar studies using Google Trends search data were conducted to predict COVID-19 infectious cases [34, 35], explore public attitudes toward vaccination [36–38], identify symptoms caused by pandemics [39–41], and assess affected medical services [42–44]. The above studies indicate that Google Trends can assist in gaining a better understanding and analysis of health information-seeking behavior. Information from Google Trends can be used to supplement current infection reports with lag times.

This study aimed to explain HFMD infections using Google Trends data from Japan before and during the COVID-19 pandemic.

## Methods

### Data

We obtained actual HFMD cases from the weekly reports issued by NIID, which included new infectious cases and sentinel cases by prefecture and updated them from 1999 to present [16]. Additionally, we set the geographic location to Japan and the category to health to limit irrelevant results and downloaded the relative search volume (RSV) of the “HFMD” search topic using Google Trends from January 1, 2009, to December 31, 2021. The normalized RSV data represent the search interest relative to the highest point for a given region and time. The scales of normalized RSV varied from 0 to 100, where 0 meant there was insufficient data for a term, and 100 was the peak popularity. We selected a search topic instead of the search term “HFMD” for comprehensive search information, and limited the period from 2009 to 2021. In this study, we used the search topic "HFMD" and the search term "HFMD." The weekly RSV of the search topic “HFMD” for each year between 2009 and 2021 were gathered for further analysis.

To identify the significant factors of HFMD infection, we created multiple linear regression models using HFMD-related search terms selected by Japanese tweets. We retrieved Japanese tweets through the publicly available Twitter Stream application programming interface (API) by querying the keywords “HFMD” to select “HFMD” related top words. Google applied improvements to the data collection system on January 1, 2016, and January 1, 2022. For consistency, 275,010 tweets restricted to 2016 and 2021 were downloaded for this study. Tokenization was used to select the top words in Japanese tweets, which is a fundamental step in many natural language processing (NLP) methods, especially for languages such as Japanese that are written without spaces between words. We tokenized all the tweets and analyzed the unigram tokens. Website links, special characters, numbers, and “amp” (ampersands) were removed from the tweets before tokenization. Python packages SpaCy and GiNZA were used to remove Japanese stop words and implement tokenization. White space characters joined tokenized words into the text in the original order. The Python package scikit-learn was used to convert the white space-joined texts into unigram tokens and calculate the token counts. The counts of tokens are provided in Additional file 1. Search terms with counts larger than 1000 and belonging to ranges of infection sources, susceptible sites, susceptible populations, symptoms, treatment, preventive measures, and identified diseases were selected for further analysis. Selected terms with corresponding interpretations and categories are provided in Additional files 2 and 3, respectively. We downloaded the weekly RSV of selected search terms through Google Trends in two periods: before (2016–2019) and during the pandemic (2020–2021) based on the first case of COVID-19 in Japan on January 16, 2020.

### Statistical analysis

First, we calculated the Pearson correlation coefficient between the HFMD cases and RSV of the search topic “HFMD” each year from 2009 to 2021 instead of the whole period due to the periodic characteristics. Because our response variable HFMD cases and explanatory variables search topic RSV were measured on a continuous scale, the parametric test was selected instead of non-parametric analysis [45].

Second, we conducted cross-correlation analyses between the HFMD cases and RSV of the selected search terms. Cross-correlation is a measure of the similarity between two series as a function of the displacement of one relative to the other, and is used to objectively estimate the time lag between cases of HFMD infection and related search terms [46]. We set the maximum lag to ± 20 weeks because of the periodic characteristics of the HFMD infection. We obtained 40 cross-correlation coefficients for each HFMD-related search term, before and during the COVID-19 pandemic. Next, we selected the coefficients with the greatest absolute values and exhibited their true values. Finally, we compared the coefficients with the highest absolute values in the periods before and during the COVID-19 pandemic. Regarding these coefficients, we assumed negative, zero, or positive values, with the greatest absolute value representing the search terms that occurred earlier, coincided with, or later than the actual HFMD cases. Differences in lags during and before the COVID-19 pandemic were calculated to determine public awareness of HFMD.

Third, we conducted multiple linear regressions to identify the most important Google search terms to explain HFMD infection before and during the COVID-19 pandemic. We included HFMD-related search terms for multiple linear regression explanatory variables with actual HFMD cases as response variables. Collinearity is the correlation between explanatory variables that express a linear relationship in a regression model. When explanatory variables are correlated in the same regression model, they cannot explain the response in a dependent manner. We normalized the RSV of each selected term to avoid collinearity in the regression models. Several common methods have been used for explanatory variable selection to identify the most significant search terms and limit the number of explanatory variables, including forward selection, backward elimination, and stepwise regression [47]. Backward elimination was used in this study to find the best subset of search terms owing to its easy implementation and automated availability. We used a p-value threshold of 0.05 to remove unnecessary search terms. In each round, we removed the search term with the highest p-value and reconstructed a multiple linear regression model until all the p-values were below the threshold.

To assess the performance of the linear regression model, the coefficients of determination R^2^ or adjusted R^2^, which indicate how much variation in response is explained by the model, are often used [48]. We selected the adjusted R^2^ value for model evaluation to avoid overfitting the model.

## Results

### Basic description of HFMD cases and “HFMD” RSV

**Fig. 1:**
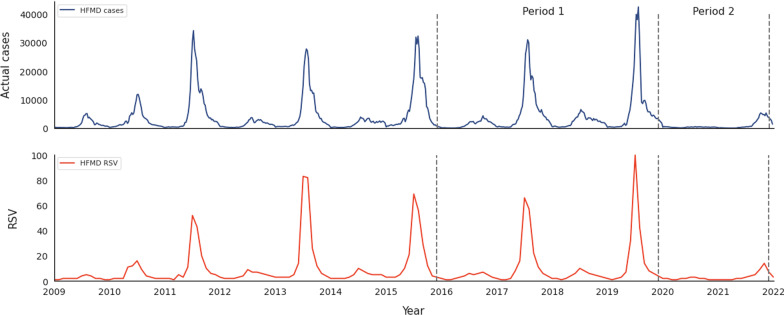
Monthly number of cases and RSV of HFMD from 2009 to 2021

Figure 1 presents the actual HFMD and RSV cases from 2009 to 2021. Visual inspection of the figure indicates that both the actual HFMD cases and RSV of Google Trends peaked around July in most years, except for 2020 and 2021. The number of HFMD infections surged after 2011, peaking every 2 years before 2020. RSV levels coincided with this trend. In 2020, no periodic peak in infection was observed, whereas in 2021, the peak was delayed to November.

**Fig. 2:**
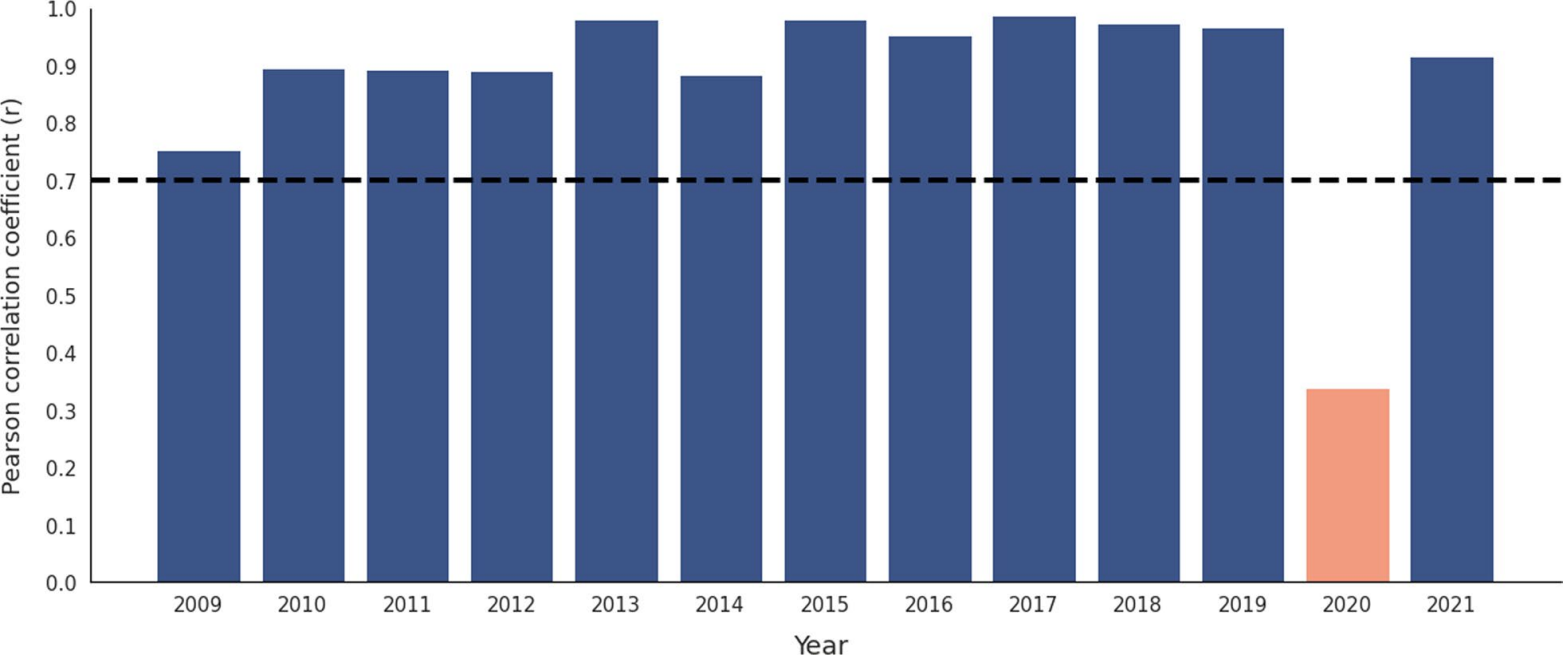
Correlation between the number of cases and RSV of HFMD for each year from 2009 to 2021

As shown in Fig. 2, we calculated the correlation between the actual HFMD cases and RSV for each year from 2009 to 2021. The mean (standard error) was 0.820 (0.052). Most coefficient values were greater than 0.7, except 0.338 in 2020. The Pearson correlation coefficients are provided in Additional file 4: Table S1.

### Cross-correlation between HFMD cases and search term RSV before and during the pandemic

We performed cross-correlation analyses to determine the temporal relationship between HFMD cases and the search term RSV. Cross-correlation results before and during the pandemic are presented in Table 1.

**Table 1:** Cross-correlation analysis of HFMD cases and search terms RSV

Search terms (English)	Search terms (Japanese)	Period 1 (2015–2019)		Period 2 (2020–2021)		Lag changes
		Lags (week)	Coefficients (r)	Lags (week)	Coefficients (r)	
/				/		Earlier
Hodgkin	ホジキン	19	0.017	− 19	0.08	− 38
Lymphoma	リンパ腫	19	0.196	− 19	0.056	− 38
Visits	受診	19	0.202	− 15	0.167	− 34
Vaccine	ワクチン	17	0.662	− 13	0.908	− 30
Fever	発熱	19	0.096	− 11	0.886	− 30
Antipyretic drug	解熱剤	19	0.177	− 11	0.926	− 30
Chickenpox	水痘	19	0.177	− 11	0.498	− 30
Reduce fever	解熱	19	0.233	− 10	0.816	− 29
Examination	検査	19	0.199	− 10	0.573	− 29
Low grade fever	微熱	19	0.07	− 9	0.178	− 28
Pediatric	小児科	17	0.529	− 6	0.639	− 23
Child (hiragana)	こども	15	0.462	− 6	0.67	− 21
Intense pain	激痛	2	0.093	− 19	0.067	− 21
Department of dermatology	皮膚科	1	0.422	− 16	0.486	− 17
Itch (word morphing)	痒く	0	0.554	− 16	0.52	− 16
Influenza	インフルエンザ	19	0.383	3	0.203	− 16
Rest	休み	2	0.428	− 14	0.363	− 16
Rash (word morphing)	プツプツ	− 2	0.478	− 17	0.325	− 15
Swimming pool	プール	0	0.625	− 15	0.699	− 15
Entero-	エンテロ	− 3	0.153	− 17	0.319	− 14
Adult	大人	− 1	0.669	− 15	0.642	− 14
Sole of the foot	足底	− 4	0.437	− 18	0.334	− 14
Nasal mucus	鼻水	18	0.326	4	0.13	− 14
Rash (word morphing)	ぶつぶつ	− 1	0.42	− 14	0.329	− 13
Rash (word morphing)	発疹	− 1	0.791	− 14	0.448	− 13
School-aged children	学童	− 3	0.124	− 16	0.059	− 13
Pharynx	咽頭	− 2	0.36	− 15	0.15	− 13
Septicemia	敗血症	12	0.338	− 1	0.109	− 13
Summer cold	夏風邪	1	0.598	− 12	0.587	− 13
Go to kindergarten	登園	− 1	0.431	− 14	0.418	− 14
Adeno-	アデノ	− 7	0.559	− 19	0.644	− 12
adenovirus	アデノウイルス	− 7	0.558	− 19	0.663	− 12
COVID-19 (short name)	コロナ	− 1	0.012	− 13	0.187	− 12
Rash (hiragana)	ボツボツ	− 1	0.227	− 13	0.208	− 12
Erythema	紅斑	− 1	0.376	− 13	0.427	− 12
Young child	小児	− 7	0.296	− 19	0.433	− 12
Kindergarten	幼稚園	− 7	0.274	− 19	0.129	− 12
Influenza (short name)	インフル	19	0.36	7	0.167	− 12
Enterovirus	エンテロウイルス	− 5	0.012	− 16	0.141	− 11
Infant	赤ちゃん	− 7	0.21	− 18	0.527	− 11
High fever	高熱	− 1	0.147	− 12	0.63	− 11
Infant (different stages)	乳幼児	− 8	0.051	− 19	0.109	− 11
Palm (colloquial term)	手のひら	− 6	0.459	− 17	0.414	− 11
Sole of the foot (colloquial term)	足裏	− 6	0.512	− 17	0.497	− 11
Daycare center	保育園	− 5	0.361	− 15	0.411	− 10
Conjunctivitis	結膜炎	− 8	0.447	− 18	0.139	− 10
Skin	皮膚	− 7	0.519	− 17	0.492	− 10
Exhaustion	疲れ	− 9	0.47	− 19	0.417	− 10
Toddler	幼児	− 7	0.069	− 17	0.491	− 10
Vomiting	嘔吐	19	0.218	9	0.224	− 10
Gastroenteritis	胃腸炎	19	0.159	9	0.404	− 10
Urticaria	蕁麻疹	− 3	0.58	− 12	0.678	− 9
palm (written term)	手掌	− 10	0.118	− 19	0.086	− 9
Swelling	腫れ	− 3	0.513	− 12	0.597	− 9
Norovirus	ノロ	19	0.11	10	0.221	− 9
Cold	風邪	18	0.403	9	0.163	− 9
Hospital	病院	− 7	0.239	− 15	0.265	− 8
Vesicle	水疱	− 1	0.595	− 8	0.668	− 7
Itch (word morphing)	痒み	− 8	0.272	− 15	0.138	− 7
Child (mixed)	子ども	− 5	0.096	− 12	0.629	− 7
Coxsackie (virus)	コクサッキー	− 2	0.216	− 8	0.123	− 6
Eczema	湿疹	− 6	0.451	− 12	0.492	− 6
Diarrhea	下痢	− 7	0.267	− 13	0.381	− 6
Chlamydia	クラミジア	5	0.133	− 1	0.226	− 6
Chickenpox (colloquial term)	水疱瘡	− 5	0.411	− 10	0.37	− 5
Herpangina	ヘルパンギーナ	0	0.575	− 4	0.716	− 4
Measles	麻疹	− 8	0.077	− 12	0.655	− 4
Papule	丘疹	− 8	0.124	− 12	0.237	− 4
Appetite	食欲	− 7	0.08	− 10	0.603	− 3
Diagnosis	診断	− 16	0.218	− 19	0.276	− 3
Child (kanji)	子供	− 11	0.248	− 14	0.489	− 3
Herpes	ヘルペス	19	0.267	17	0.094	− 2
Herpes	疱疹	− 1	0.385	− 2	0.815	− 1

/				/		Coincide
Oral cavity (synonym)	口腔	− 19	0.248	− 19	0.189	0
Aphtha	アフタ	9	0.143	9	0.143	0
Gargle	うがい	19	0.4	19	0.005	0
Disinfection	消毒	19	0.078	19	0.13	0
Company	会社	19	0.183	19	0.059	19

/				/		Later
Hand and foot	手足	− 1	0.976	0	0.842	1
Pneumonia	肺炎	18	0.099	19	0.128	1
School	学校	18	0.068	19	0.051	19
Pain (word morphing)	痛い	− 10	0.305	− 8	0.738	2
Mycoplasma	マイコプラズマ	17	− 0.031	19	0.143	2
Stomach	お腹	− 7	0.316	− 4	0.493	3
Handwashing	手洗い	15	0.255	19	0.133	4
Allergy	アレルギー	− 19	0.42	− 13	0.412	6
Itch(hiragana)	かゆい	− 19	0.371	− 12	0.41	7
Pain (word morphing)	痛み	− 19	0.203	− 10	0.309	9
Pain (word morphing)	痛く	− 19	0.245	− 8	0.313	11
Headache	頭痛	− 19	0.032	− 8	0.535	11
Stomatitis	口内炎	0	0.52	19	0.05	19
Vesicle (colloquial term)	水泡	0	0.678	19	− 0.002	19
Meningitis	髄膜炎	0	0.304	19	0.058	19
Typhus	チフス	− 4	0.084	19	0.054	23
Rubella	風疹	− 6	0.017	19	0.086	25
Hemolytic streptococcus	溶連菌	− 7	0.355	19	0.132	26
Infant (different stages)	乳児	− 7	0.107	19	0.083	26
Legionella	レジオネラ	− 18	0.282	9	0.064	27
Virus	ウイルス	− 9	0.207	19	0.125	28
Oral cavity (synonym)	口内	− 19	0.084	9	0.054	28
Specific medicine	特効薬	− 19	0.129	19	0.11	38

Compared with period 1, the temporal correlation between HFMD cases and the search term RSV changed in period 2. In period 1, 61 (60.4%), 6 (5.9%), and 34 (33.7%) of the search terms RSV presented earlier, coincided with, and later than the HFMD cases, respectively. In period 2, 73 (72.3%), 1 (1%), and 27 (26.7%) presented earlier, coincided with, and later, respectively, than the HFMD cases. The differences in lags for 73 (72.3%) search terms were negative, which might indicate increasing public awareness of HFMD infections during the COVID-19 pandemic. In contrast, lags for the five search terms did not change, and 23 search terms exhibited delays.

### Essential search terms for explaining HFMD cases before and during the pandemic

We identified the most significant search terms for HFMD infection using multiple linear regression with backward elimination. As shown in Table 2, 18 search terms were significant in period 1 and accounted for an adjusted R^2^ value of 96.7% of the variation in HFMD infection. Conversely, as shown in Table 3, 57 search terms were detected as significant, with adjusted R^2^ = 98.4%, in period 2, which included more critical variables than in period 1. The model specification formulas are provided in Additional file 4: Table S2 and Table S3.

**Table 2:** Multiple linear regression model of HFMD cases and RSV of selected search terms from 2016 to 2019

Search terms (English)	Search terms (Japanese)	Model summary		
		Standardized β	Std.Error	P-value
Virus	ウイルス	0.0507	0.019	0.007
Herpangina	ヘルパンギーナ	− 0.1034	0.033	0.002
Diarrhea	下痢	− 0.0998	0.021	< 0.001
Oral cavity (synonym)	口内	0.0290	0.015	0.049
Summer cold	夏風邪	0.1361	0.037	< .001
Adult	大人	0.1306	0.032	< .001
Young child	小児	− 0.0372	0.017	0.032
Pediatric	小児科	0.0641	0.019	0.001
Hand and foot	手足	0.8575	0.026	< 0.001
Chickenpox (colloquial term)	水疱瘡	− 0.0634	0.018	0.001
Eczema	湿疹	0.0506	0.018	0.006
Intense pain	激痛	0.0361	0.014	0.010
Exhaustion	疲れ	− 0.0474	0.021	0.024
Itch (word morphing)	痒く	0.0468	0.019	0.014
Swelling	腫れ	− 0.0481	0.022	0.033
Reduce fever	解熱	− 0.1042	0.021	< 0.001
Sole of the foot	足底	− 0.0589	0.023	0.012
Nasal mucus	鼻水	− 0.0743	0.023	0.001

Adjusted R^2^ = 96.7%

**Table 3:** Multiple linear regression of HFMD cases and RSV of selected search terms between 2020 and 2021

Search terms (English)	Search terms (Japanese)	Model summary		
		Standardized β	Std.Error	P-value
Adenovirus	アデノウイルス	− 0.2816	0.04	< 0.001
Influenza (short name)	インフル	− 0.1938	0.062	0.003
Entero-	エンテロ	0.1566	0.021	< 0.001
Stomach	お腹	0.0897	0.028	0.002
Coxsackie (virus)	コクサッキー	0.0446	0.02	0.034
Child (hiragana)	こども	− 0.4402	0.111	< 0.001
COVID-19 (short name)	コロナ	0.6273	0.102	< 0.001
Rash (word morphing)	プツプツ	0.1868	0.029	< 0.001
Herpangina	ヘルパンギーナ	0.8604	0.044	< 0.001
Herpes	ヘルペス	− 0.3042	0.042	< 0.001
Hodgkin	ホジキン	0.0464	0.017	0.009
Mycoplasma	マイコプラズマ	0.1905	0.069	0.008
Legionella	レジオネラ	0.1888	0.025	< 0.001
Vaccine	ワクチン	− 1.1388	0.136	< 0.001
Diarrhea	下痢	0.2803	0.056	< 0.001
Papule	丘疹	0.0863	0.02	< 0.001
Infant (different stages)	乳児	− 0.2984	0.034	< 0.001
Infant (different stages)	乳幼児	0.0712	0.024	0.005
Daycare center	保育園	0.3336	0.049	< 0.001
Visits	受診	0.1898	0.038	< 0.001
Oral cavity (synonym)	口腔	0.1055	0.034	0.004
Pharynx	咽頭	0.1182	0.022	< 0.001
Vomiting	嘔吐	− 0.0955	0.046	0.045
Summer cold	夏風邪	− 0.9272	0.061	< 0.001
Child (mixed)	子ども	0.1727	0.045	< 0.001
Child (kanji)	子供	0.2808	0.041	< 0.001
Young child	小児	− 0.2281	0.049	< 0.001
Pediatric	小児科	0.6066	0.114	< 0.001
Toddler	幼児	0.1418	0.045	0.003
Kindergarten	幼稚園	− 0.2225	0.043	< 0.001
Palm (colloquial term)	手のひら	0.1388	0.029	< 0.001
Handwashing	手洗い	− 0.1137	0.051	0.031
Septicemia	敗血症	0.1228	0.017	< 0.001
Vesicle (colloquial term)	水泡	− 0.2038	0.037	< 0.001
Vesicle	水疱	0.1718	0.035	< 0.001
Chickenpox	水痘	0.0902	0.021	< 0.001
Disinfection	消毒	− 0.4493	0.082	< 0.001
Hemolytic streptococcus	溶連菌	0.3638	0.086	< 0.001
Specific medicine	特効薬	− 0.4039	0.047	< 0.001
Herpes	疱疹	0.1362	0.042	0.002
Exhaustion	疲れ	− 0.3952	0.041	< 0.001
Hospital	病院	− 0.214	0.05	< 0.001
Itch (word morphing)	痒み	0.1993	0.032	< 0.001
Pain (word morphing)	痛い	− 0.2462	0.047	< 0.001
Pain (word morphing)	痛く	− 0.2206	0.029	< 0.001
Rash (word morphing)	発疹	− 0.2028	0.055	0.001
Skin	皮膚	0.1754	0.04	< 0.001
Conjunctivitis	結膜炎	− 0.1782	0.035	< 0.001
Pneumonia	肺炎	0.3648	0.06	< 0.001
Swelling	腫れ	0.152	0.043	0.001
Urticaria	蕁麻疹	0.747	0.147	< 0.001
Antipyretic drug	解熱剤	0.3263	0.105	0.003
Infant	赤ちゃん	0.2101	0.035	< 0.001
Sole of the foot (colloquial term)	足裏	− 0.2455	0.052	< 0.001
High fever	高熱	0.1784	0.07	0.014
Measles	麻疹	− 0.5895	0.126	< 0.001
Nasal mucus	鼻水	− 0.2792	0.05	< 0.001

Adjusted R^2^ = 98.4%

Compared to period 1, the significant search terms in period 2 contained the same meanings and expanded informative search content. “Herpangina,” “nasal mucus,” “exhaustion,” “diarrhea,” “summer cold,” “young child,” “pediatric,” and “swelling” occurred in the original form. “Pain,” “reduce fever,” “oral cavity,” “chickenpox,” “itch,” and “sole of the foot” occurred in morphing or synonym forms. “Virus” was replaced by more specific infection sources in period 2, such as “adenovirus,” “entero-,” “coxsackie,” “mycoplasma,” “legionella,” and “hemolytic streptococcus.” Correspondingly, “adult” was superseded by other terms for susceptible populations, such as “child” and “infant” (Omit synonyms). Search terms related to susceptible sites, preventive measures, and treatment also were identified in period 2, such as “daycare center,” “kindergarten,” “handwashing,” “disinfection,” “hospital,” and “specialty drugs.” Multiple linear regression results corroborated the cross-correlation results and indicated that public awareness of HFMD might increase during the COVID-19 pandemic.

## Discussion

This study presented trends and correlations between HFMD cases and RSV of “HFMD” from 2009 to 2021. Cross-correlation analyses were conducted between HFMD cases and search terms for RSV before and during the pandemic period. Additionally, multiple linear regressions were used to identify significant search terms for explaining HFMD cases in the two periods. Our results indicated that HFMD cases and RSV peaked around July in most years, except in 2020 and 2021, and surged after 2011 with peaks every 2 years before 2020. The search topic “HFMD” exhibited strong correlations with HFMD cases except in 2020, when the COVID-19 outbreak occurred. Furthermore, cross-correlation and multiple regression results revealed that the public might have improved their awareness of HFMD infection during the pandemic. To our knowledge, this study is the first to explain HFMD cases using Google search data and to examine changes in information-seeking behavior toward HFMD affected by the COVID-19 pandemic. Google search data can supplement public health surveillance and help authorities respond rapidly to infectious diseases.

From 2009 to 2021, the RSV of “HFMD” coincided with the HFMD cases, except in 2020, which showed similar trends and peaks. In Japan, HFMD peaks generally occur in July [5]. During the COVID-19 pandemic, different from previous HFMD peaks disappeared in 2020 and lagged until November 2021. In 2020, Google Trends search data did not match the “HFMD” cases, with a relatively small peak in July. Despite a small peak in the RSV of the search topic “HFMD,” the volume was much lower than in previous years. In contrast, the Japanese government has implemented several measures to control COVID-19, which might potentially influence the spread of HFMD. Respiratory droplets and contact routes are the main infection routes in HFMD and COVID-19 [49, 50]. Therefore, the population susceptible to HFMD also stays safe by taking standard precautions during COVID-19, such as physical distancing, wearing a mask, regularly washing hands, and coughing into a bent elbow or tissue [51].

Based on the evidence provided by our results, the global pandemic might have enhanced public awareness of HFMD in addition to COVID-19. 73 (72.3%) search terms cross-correlated earlier with HFMD cases during COVID-19, and significant search terms detected in Period 2 contained more informative information. Previous studies demonstrated that the prevalence of respiratory infectious diseases reduced during the COVID-19 pandemic, such as influenza, varicella, herpes zoster, rubella, and measles [52–58]. This might have been due to adherence to non-pharmaceutical interventions and lower non-polio enterovirus activity during the COVID-19 pandemic compared to 2014–2019 [59]. Switzerland had an unprecedented complete absence of pediatric enteroviral meningitis in 2020 [60]. In Japan, community-acquired pneumonia [61] and influenza [62] admissions have reduced during the COVID-19 pandemic. COVID-19 preventative actions and better personal hygiene are beneficial for preventing the spread of the disease. However, the prevalence of common diseases may rise as the public gradually complies less with infection control measures in the upcoming season [61]. Consistent with our results, a peak in HFMD infection and public interest reoccurred in November 2021. Continuous monitoring of HFMD is required, and public information-seeking behaviors may be helpful in public health surveillance.

Google search data was applied in our study to explain HFMD cases affected by COVID-19 instead of Baidu search data, which was used for real-time estimation of HFMD cases in China [15] and other categories of data for HFMD prediction or explanation [11–14]. In contrast to previous studies, we focused on the distinction between the information-seeking behavior of HFMD before and during COVID-19 and attempted to explain the HFMD cases using Google Trends data, which has the highest market share in Japan [17]. Many researchers have shown that Google search data represent public interest in a specific topic. However, Google search data should be used cautiously as a surveillance system, because large events can easily interfere with it. Combining fine-grained data, such as mobility data, could help develop surveillance systems that can effectively exclude biased or irrelevant information to respond rapidly [63].

## Conclusion

This study described trends and correlations in HFMD cases with RSV of “HFMD” and identified significant search terms to explain HFMD infections before and during the COVID-19 pandemic. We found that the prevalence of HFMD was abnormal during COVID-19, and the public might have enhanced awareness of HFMD infection affected by the pandemic. It is critical to continuously monitor resurgent common infections, as the public gradually reduces compliance with infection control measures. Public information seeking behavior using Google search data may be useful for public health surveillance.

## Limitations

This study has several limitations. First, our findings are limited to those who used Google to search for health-related information, which may not represent the entire community. The results may be biased toward younger people, who are more digitally connected than older individuals, although the Google search engine market share in Japan is nearly 80% [17]. Second, Google Trends improved the geographical assignment and data collection systems in 2011, 2016, and 2022. Our results in the basic description of HFMD cases and “HFMD” RSV might be affected by them. Third, search data analysis is hypersensitive to large events; therefore, it is complementary instead of replacing traditional research methods. Fourth, the specific HFMD-related terms selected by Twitter may not represent all search terms in public use, especially hiragana, katakana, kanji, and alphabets used in Japan. Fifth, we used search data from 2016 to 2019 to represent the period before the COVID-19 pandemic owing to restrictions of Google Trends, which may not represent the entire period. Finally, during the process of backward elimination for regression model construction, significant terms may be eliminated because they are jointly insignificant. Although we can find potentially significant terms from all eliminated terms, it is not practical to conduct F-tests multiple times because multiple hypothesis testing leads to lower confidence levels. Hence, the current processing results were retained.

## Supplementary Information

**Additional file 1.** Counts of tokens calculated using "HFMD" related Tweets.

**Additional file 2.** Translation tables and interpretations.

**Additional file 3.** Search terms categories.

**Additional file 4.** Cross-correlation coefficients and model specification formulas.

**Additional file 5.** Raw data.

## Data Availability

We used publicly available data published by Google Trends (https://trends.google.com/trends/?geo=JP) and the National Institute of Infectious Diseases, Japan (https://www.niid.go.jp/niid/en/). All data generated or analyzed during this study are included in the published article (Additional file 5).

## Abbreviations

HFMD: Hand, foot, and mouth disease
EV-A71: Enterovirus A71
CV-A6: Coxsackievirus A6
CV-A16: Coxsackievirus A16
NIID: National Institute of Infectious Disease
LDE: Logistic differential equation
L-DNM: Landscape dynamic network marker
COPD: Chronic obstructive pulmonary disease
RSV: Relative search volume
