# Supplementary material for: Explanation of hand, foot, and mouth disease cases in Japan using Google Trends before and during the COVID-19: infodemiology study
Source: BMC Infect Dis. 2022 Oct 29;22:806. doi: 10.1186/s12879-022-07790-9 (PMC9617033; doi:10.1186/s12879-022-07790-9)
Supplement: Supplementary file 4 — Additional file 4. Cross-correlation coefficients and model specification formulas. [file 12879_2022_7790_MOESM4_ESM.docx]

**Legend:**

Pearson correlations were calculated between the actual HFMD cases and RSV for each year from 2009 to 2021. The mean (standard error) was 0.820 (0.052). Most coefficient values were greater than 0.7, except 0.338 in 2020. The correlation coefficients are in Table-S1.

The most significant search terms were identified for HFMD infection using multiple linear regression with backward elimination. The model specification formulas are in Table-S2 and Table-S3.

**Table-S1.** Pearson correlation coefficients between HFMD cases and RSV of “HFMD”

| Years | Pearson correlation coefficients |
| --- | --- |
| 2009 | 0.753 |
| 2010 | 0.896 |
| 2011 | 0.894 |
| 2012 | 0.891 |
| 2013 | 0.980 |
| 2014 | 0.883 |
| 2015 | 0.981 |
| 2016 | 0.952 |
| 2017 | 0.988 |
| 2018 | 0.974 |
| 2019 | 0.966 |
| 2020 | 0.338 |
| 2021 | 0.916 |
| 1. Coefficients values under than 0.7 were marked in red. | |

**Table-S2** Ordinary Least Squares (OLS) Regression Results for 2016-2019

| **Response variable:** | | HFMD cases | | **R-squared:** | | 0.970 |
| --- | --- | --- | --- | --- | --- | --- |
| **Explanatory variable:** | | Search terms RSV | | **Adj. R-squared:** | | 0.967 |
| **Model:** | | OLS | | **F-statistic:** | | 338.0 |
| **Method:** | | Least Squares | | **Prob (F-statistic):** | | 3.34e-133 |
| **Date:** | | Tue, 20 Sep 2022 | | **Log-Likelihood:** | | 69.583 |
| **Time:** | | 15:32:43 | | **AIC:** | | -101.2 |
| **No. Observations:** | | 208 | | **BIC:** | | -37.75 |
| **Df Residuals:** | | 189 | |  | |  |
| **Df Model:** | | 18 | |  |  |  |
| **Covariance Type:** | | nonrobust | |  |  |  |
| **Search terms (English)** | **Search terms**  **(Japanese)** | **Standardized β** | **Std.Error** | **P-value** | **[0.025** | **0.975]** |
| virus | ウイルス | 0.0507 | 0.019 | 0.007 | 0.014 | 0.088 |
| herpangina | ヘルパンギーナ | -0.1034 | 0.033 | 0.002 | -0.168 | -0.039 |
| diarrhea | 下痢 | -0.0998 | 0.021 | <0.001 | -0.141 | -0.059 |
| oral cavity (synonym) | 口内 | 0.0290 | 0.015 | 0.049 | 9.94E-05 | 0.058 |
| summer cold | 夏風邪 | 0.1361 | 0.037 | <.001 | 0.063 | 0.209 |
| adult | 大人 | 0.1306 | 0.032 | <.001 | 0.068 | 0.194 |
| young child | 小児 | -0.0372 | 0.017 | 0.032 | -0.071 | -0.003 |
| pediatric | 小児科 | 0.0641 | 0.019 | 0.001 | 0.026 | 0.102 |
| hand and foot | 手足 | 0.8575 | 0.026 | <0.001 | 0.806 | 0.909 |
| chickenpox (colloquial term) | 水疱瘡 | -0.0634 | 0.018 | 0.001 | -0.1 | -0.027 |
| eczema | 湿疹 | 0.0506 | 0.018 | 0.006 | 0.015 | 0.086 |
| intense pain | 激痛 | 0.0361 | 0.014 | 0.010 | 0.009 | 0.064 |
| exhaustion | 疲れ | -0.0474 | 0.021 | 0.024 | -0.088 | -0.006 |
| itch (word morphing) | 痒く | 0.0468 | 0.019 | 0.014 | 0.01 | 0.084 |
| swelling | 腫れ | -0.0481 | 0.022 | 0.033 | -0.092 | -0.004 |
| reduce fever | 解熱 | -0.1042 | 0.021 | <0.001 | -0.145 | -0.064 |
| sole of the foot | 足底 | -0.0589 | 0.023 | 0.012 | -0.105 | -0.013 |
| nasal mucus | 鼻水 | -0.0743 | 0.023 | 0.001 | -0.119 | -0.03 |
| **Omnibus:** | | 47.436 |  | **Durbin-Watson:** | | 1.290 |
| **Prob (Omnibus):** | | 0.000 |  | **Jarque-Bera (JB):** | | 512.343 |
| **Skew:** | | -0.417 |  | **Prob (JB):** | | 5.57e-112 |
| **Kurtosis:** | | 10.643 |  | **Cond. No.** | | 9.21 |
| Notes:  [1] Standard Errors assume that the covariance matrix of the errors is correctly specified. | | | | | | |

**Table-S3** Ordinary Least Squares (OLS) Regression Results for 2020-2021

| **Response variable:** | | HFMD cases | | **R-squared:** | | 0.993 |
| --- | --- | --- | --- | --- | --- | --- |
| **Explanatory variable:** | | Search terms RSV | | **Adj. R-squared:** | | 0.984 |
| **Model:** | | OLS | | **F-statistic:** | | 113.5 |
| **Method:** | | Least Squares | | **Prob (F-statistic):** | | 4.07e-36 |
| **Date:** | | Wed, 21 Sep 2022 | | **Log-Likelihood:** | | 110.49 |
| **Time:** | | 10:57:08 | | **AIC:** | | -105.0 |
| **No. Observations:** | | 104 | | **BIC:** | | 48.40 |
| **Df Residuals:** | | 46 | |  | |  |
| **Df Model:** | | 57 | |  | |  |
| **Covariance Type:** | | nonrobust | |  | |  |
| **Search terms (English)** | **Search terms**  **(Japanese)** | **Standardized β** | **Std.Error** | **P-value** | **[0.025** | **0.975]** |
| adenovirus | アデノウイルス | -0.2816 | 0.04 | <0.001 | -0.362 | -0.201 |
| influenza (short name) | インフル | -0.1938 | 0.062 | 0.003 | -0.319 | -0.069 |
| entero- | エンテロ | 0.1566 | 0.021 | <0.001 | 0.114 | 0.2 |
| stomach | お腹 | 0.0897 | 0.028 | 0.002 | 0.034 | 0.145 |
| coxsackie (virus) | コクサッキー | 0.0446 | 0.02 | 0.034 | 0.004 | 0.086 |
| child (hiragana) | こども | -0.4402 | 0.111 | <0.001 | -0.664 | -0.217 |
| COVID-19 (short name) | コロナ | 0.6273 | 0.102 | <0.001 | 0.422 | 0.833 |
| rash (word morphing) | プツプツ | 0.1868 | 0.029 | <0.001 | 0.129 | 0.245 |
| herpangina | ヘルパンギーナ | 0.8604 | 0.044 | <0.001 | 0.772 | 0.949 |
| herpes | ヘルペス | -0.3042 | 0.042 | <0.001 | -0.389 | -0.22 |
| hodgkin | ホジキン | 0.0464 | 0.017 | 0.009 | 0.012 | 0.081 |
| mycoplasma | マイコプラズマ | 0.1905 | 0.069 | 0.008 | 0.051 | 0.33 |
| legionella | レジオネラ | 0.1888 | 0.025 | <0.001 | 0.139 | 0.239 |
| vaccine | ワクチン | -1.1388 | 0.136 | <0.001 | -1.412 | -0.866 |
| diarrhea | 下痢 | 0.2803 | 0.056 | <0.001 | 0.168 | 0.392 |
| papule | 丘疹 | 0.0863 | 0.02 | <0.001 | 0.047 | 0.126 |
| infant (different stages) | 乳児 | -0.2984 | 0.034 | <0.001 | -0.367 | -0.23 |
| infant (different stages) | 乳幼児 | 0.0712 | 0.024 | 0.005 | 0.023 | 0.12 |
| daycare center | 保育園 | 0.3336 | 0.049 | <0.001 | 0.234 | 0.433 |
| visits | 受診 | 0.1898 | 0.038 | <0.001 | 0.114 | 0.266 |
| oral cavity (synonym) | 口腔 | 0.1055 | 0.034 | 0.004 | 0.036 | 0.175 |
| pharynx | 咽頭 | 0.1182 | 0.022 | <0.001 | 0.075 | 0.162 |
| vomiting | 嘔吐 | -0.0955 | 0.046 | 0.045 | -0.189 | -0.002 |
| summer cold | 夏風邪 | -0.9272 | 0.061 | <0.001 | -1.05 | -0.804 |
| child (mixed) | 子ども | 0.1727 | 0.045 | <0.001 | 0.082 | 0.264 |
| child (kanji) | 子供 | 0.2808 | 0.041 | <0.001 | 0.197 | 0.364 |
| young child | 小児 | -0.2281 | 0.049 | <0.001 | -0.327 | -0.129 |
| pediatric | 小児科 | 0.6066 | 0.114 | <0.001 | 0.376 | 0.837 |
| toddler | 幼児 | 0.1418 | 0.045 | 0.003 | 0.052 | 0.231 |
| kindergarten | 幼稚園 | -0.2225 | 0.043 | <0.001 | -0.309 | -0.136 |
| palm (colloquial term) | 手のひら | 0.1388 | 0.029 | <0.001 | 0.08 | 0.198 |
| handwashing | 手洗い | -0.1137 | 0.051 | 0.031 | -0.217 | -0.011 |
| septicemia | 敗血症 | 0.1228 | 0.017 | <0.001 | 0.088 | 0.158 |
| vesicle (colloquial term) | 水泡 | -0.2038 | 0.037 | <0.001 | -0.279 | -0.129 |
| vesicle | 水疱 | 0.1718 | 0.035 | <0.001 | 0.101 | 0.243 |
| chickenpox | 水痘 | 0.0902 | 0.021 | <0.001 | 0.047 | 0.133 |
| disinfection | 消毒 | -0.4493 | 0.082 | <0.001 | -0.615 | -0.283 |
| hemolytic streptococcus | 溶連菌 | 0.3638 | 0.086 | <0.001 | 0.192 | 0.536 |
| specific medicine | 特効薬 | -0.4039 | 0.047 | <0.001 | -0.499 | -0.309 |
| herpes | 疱疹 | 0.1362 | 0.042 | 0.002 | 0.053 | 0.22 |
| exhaustion | 疲れ | -0.3952 | 0.041 | <0.001 | -0.478 | -0.312 |
| hospital | 病院 | -0.214 | 0.05 | <0.001 | -0.315 | -0.113 |
| itch (word morphing) | 痒み | 0.1993 | 0.032 | <0.001 | 0.134 | 0.265 |
| pain (word morphing) | 痛い | -0.2462 | 0.047 | <0.001 | -0.341 | -0.151 |
| pain (word morphing) | 痛く | -0.2206 | 0.029 | <0.001 | -0.279 | -0.162 |
| rash (word morphing) | 発疹 | -0.2028 | 0.055 | 0.001 | -0.314 | -0.091 |
| skin | 皮膚 | 0.1754 | 0.04 | <0.001 | 0.096 | 0.255 |
| conjunctivitis | 結膜炎 | -0.1782 | 0.035 | <0.001 | -0.248 | -0.108 |
| pneumonia | 肺炎 | 0.3648 | 0.06 | <0.001 | 0.243 | 0.486 |
| swelling | 腫れ | 0.152 | 0.043 | 0.001 | 0.066 | 0.238 |
| urticaria | 蕁麻疹 | 0.747 | 0.147 | <0.001 | 0.451 | 1.043 |
| antipyretic drug | 解熱剤 | 0.3263 | 0.105 | 0.003 | 0.116 | 0.537 |
| infant | 赤ちゃん | 0.2101 | 0.035 | <0.001 | 0.139 | 0.281 |
| sole of the foot (colloquial term) | 足裏 | -0.2455 | 0.052 | <0.001 | -0.351 | -0.14 |
| high fever | 高熱 | 0.1784 | 0.07 | 0.014 | 0.038 | 0.319 |
| measles | 麻疹 | -0.5895 | 0.126 | <0.001 | -0.843 | -0.336 |
| nasal mucus | 鼻水 | -0.2792 | 0.05 | <0.001 | -0.38 | -0.178 |
| **Omnibus:** | | 5.460 | | **Durbin-Watson:** | | 2.472 |
| **Prob (Omnibus):** | | 0.065 | | **Jarque-Bera (JB):** | | 5.291 |
| **Skew:** | | 0.371 | | **Prob (JB):** | | 0.0710 |
| **Kurtosis:** | | 3.818 | | **Cond. No.** | | 67.5 |
| Notes:  [1] Standard Errors assume that the covariance matrix of the errors is correctly specified. | | | | | | |
